# Uremic lion face syndrome

**DOI:** 10.1590/2175-8239-JBN-2018-0198

**Published:** 2019-01-21

**Authors:** Joana Gameiro, Inês Duarte, Cristina Outerelo, José António Lopes

**Affiliations:** 1 Centro Hospitalar Lisboa Norte EPE Serviço de Nefrologia e Transplantação Renal Lisboa Portugal Centro Hospitalar Lisboa Norte EPE, Serviço de Nefrologia e Transplantação Renal, Lisboa, Portugal.

**Keywords:** Hyperparathyroidism, Secondary, Hyperostosis Frontalis Interna, Fractures, Bone, Renal Insufficiency, Chronic

## Abstract

Mineral bone disorder is a common feature of chronic kidney disease. Lion face syndrome is rare complication of severe hyperparathyroidism in end-stage renal disease patients, which has been less commonly reported due to dialysis and medical treatment advances in the last decade. The early recognition of the characteristic facial deformity is crucial to prompt management and prevent severe disfigurement. The authors present a rare case of severe hyperparathyroidism presenting with lion face syndrome and bone fractures.

## Background

Mineral bone disorder is a common feature of chronic kidney disease. It is mainly consequent of secondary hyperparathyroidism, which develops as an adaptive response to phosphorus retention.^1^ Severe secondary hyperparathyroidism is associated with bone deformities, increased bone fracture risk, cardiovascular events, and mortality risk. Parathyroidectomy is required in patients with persistently elevated parathyroid hormone (PTH) refractory to medical treatment, improving patient outcomes.^1^

The authors present a rare case of severe hyperparathyroidism presenting with lion face syndrome and bone fractures.

## Case report

**Figure 1:**
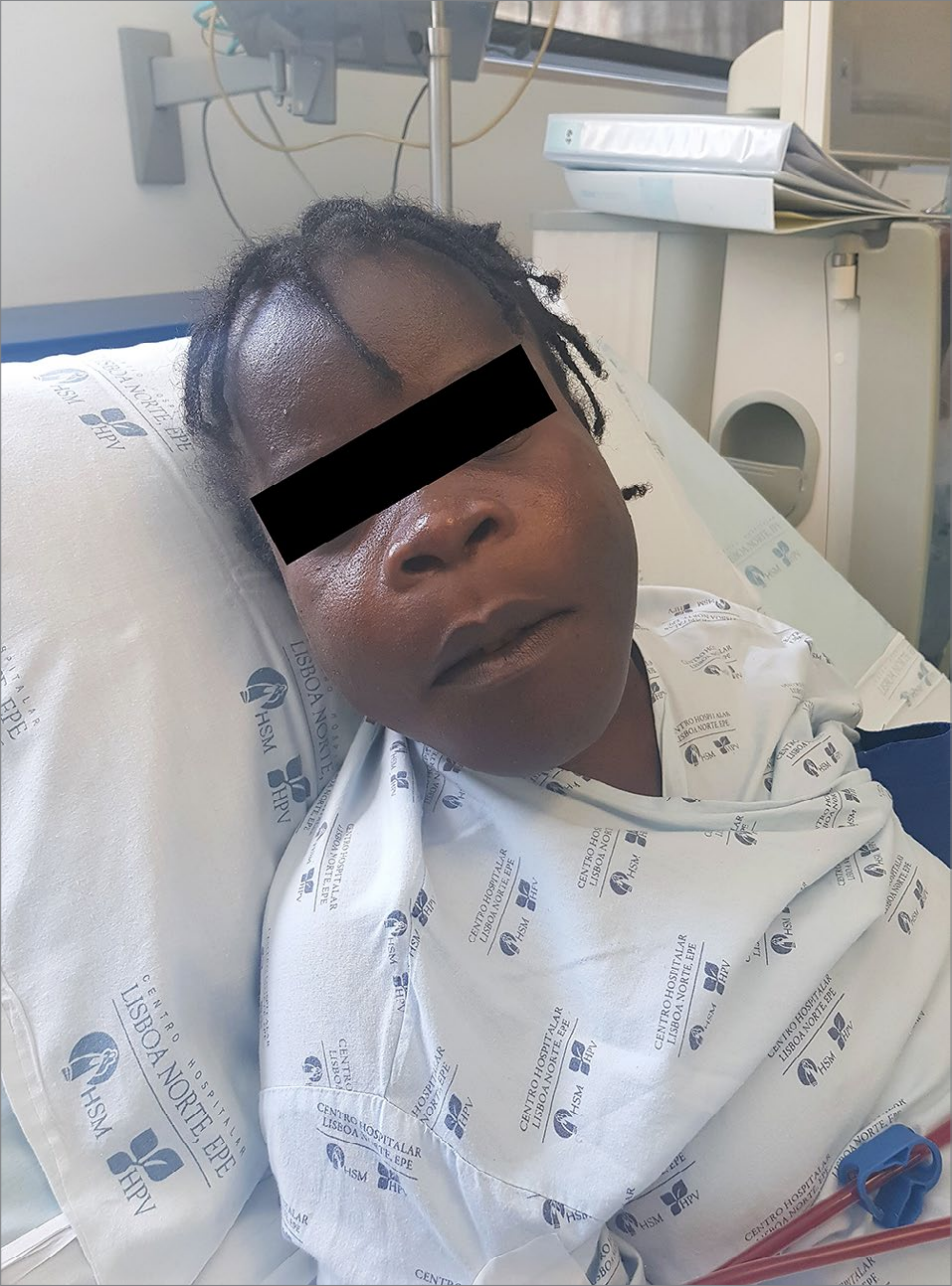
Patient's physical examination

A 39-year-old female with end-stage renal disease on hemodialysis for 11 years was transferred from Angola to Portugal for an orthopedic consultation due to multiple pathological fractures in the lower limbs. In the previous year, she noticed progressive bone pain, difficulty in walking, and facial deformation. She was medicated only with anti-hypertensive medication and erythropoiesis stimulating agent in high dose. On the physical examination, she had maxillary hypertrophy, flattened nose and spread dentition, with a 'lion face' appearance (Figure 1). She had deformed lower limbs and was unable to walk. Laboratory findings showed hemoglobin of 8.0 g/dL, serum calcium of 9.6 mg/dL, serum phosphorus of 3.3 mg/dL, and serum PTH concentration of 4302 pg/mL. Ultrasound revealed hyperplasia of the parathyroid gland. Her head CT showed thickening of cranial bones, widening of the diploic space of the skull with sclerotic and lytic changes and overgrowths in the mandible with ground glass appearance (Figure S1). Pelvic radiography demonstrated altered bone density, basicervical right femoral fracture, and left consolidated subtrochanteric femoral fracture (Figure S2). The diagnosis of severe complicated secondary hyperparathyroidism was confirmed. The patient was unresponsive to calcimimetics and vitamin D analogs and underwent parathyroidectomy.

The post-operative course was complicated with severe hungry bone syndrome, characterized by severe hypocalcemia (serum calcium of 4.6 mg/dL), which was only managed with a combination of calcium reposition, teriparatide and paricalcitol. Her clinical condition remained stable and facial deterioration stopped.

## Discussion

Lion face syndrome or leontiasis ossea is a rare complication of severe hyperparathyroidism in end-stage renal disease patients, which has been less commonly reported due to dialysis and medical treatment advances in the last decade.^2^ This condition is characterized by hyperostotic changes in the facial bones that can result in bilateral expansion of the malar processes.^3^ Therapeutic management is limited. Parathyroidectomy ceases further facial deterioration although, as demonstrated by our case, it may be complicated by hungry bone syndrome. Surgical correction of facial bones is controversial and facial deterioration can stabilize or improve mildly after parathyroidectomy, which highlights the importance of early recognition to prompt management and prevent severe disfigurement.^4^ This highlights the importance of recognizing the distinctive phenotype of this rare complication.

## Supplementary material

The following online material is available for this article:

Figure S1. Patient's head CT.

Figure S2. Pelvic radiography.
