# Supplementary figures and images for: Uremic lion face syndrome
Source: J Bras Nefrol. 2019 Jan 21;41(2):304–5. doi: 10.1590/2175-8239-JBN-2018-0198 (PMC6699430; doi:10.1590/2175-8239-JBN-2018-0198)

## Supplementary Material to “Uremic lion face syndrome”

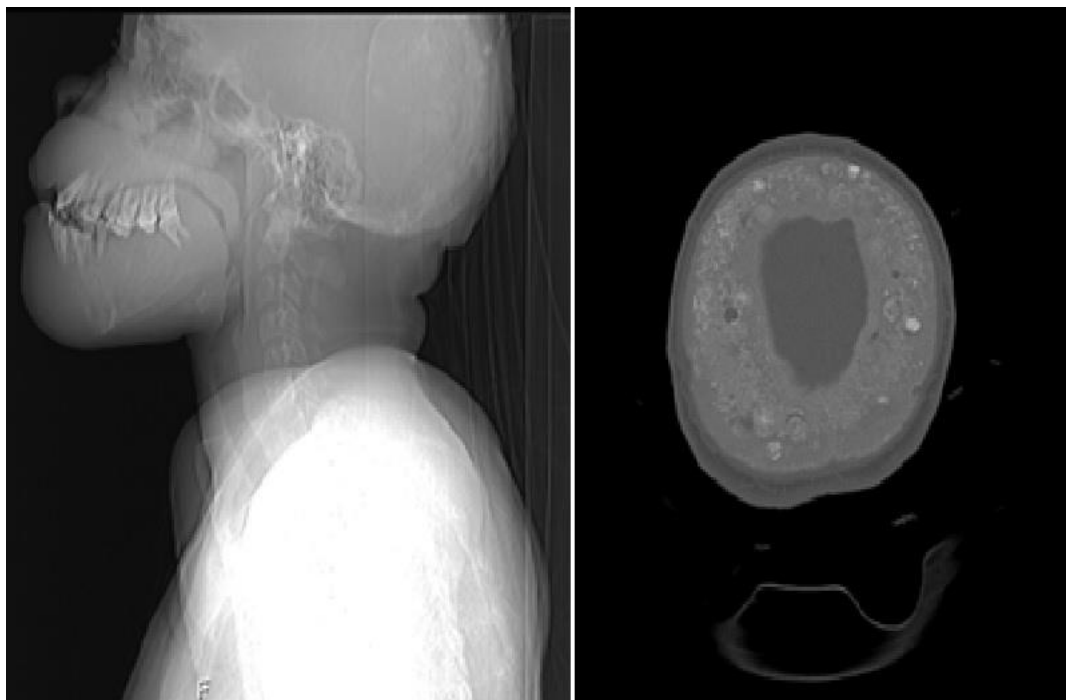

**Figure S1.** Patient's head CT.

Supplement: Supplementary file 1 [file 2175-8239-jbn-2018-0198-suppl01.pdf]

## Supplementary Material to “Uremic lion face syndrome”

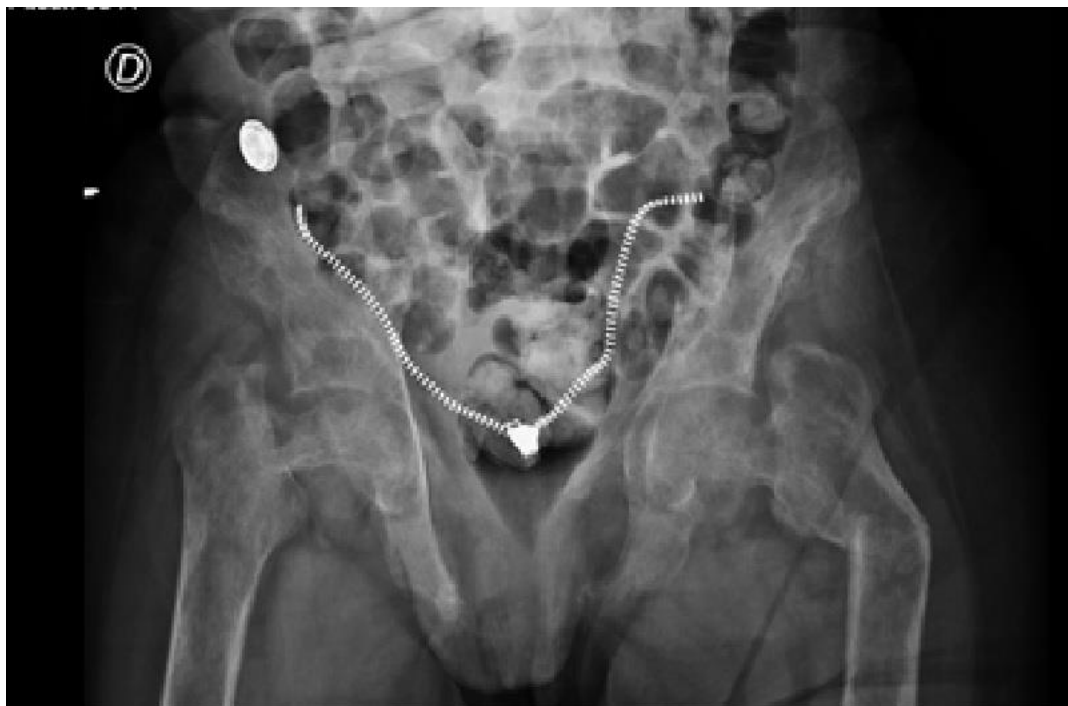

**Figure S2.** Pelvic radiography.

Supplement: Supplementary file 2 [file 2175-8239-jbn-2018-0198-suppl02.pdf]
